# Supplementary material for: Music Listening, Emotion, and Cognition in Older Adults
Source: Brain Sci. 2022 Nov 17;12(11):1567. doi: 10.3390/brainsci12111567 (PMC9688894; doi:10.3390/brainsci12111567)
Supplement: Supplementary file 1 [file brainsci-12-01567-s001.zip › brainsci-2019018-supplementary.pdf]

SUPPLEMENTARY MATERIALS
Music Listening, Emotion, and Cognition in Older Adults

Table S1. Statistical Comparisons of the Six Groups of Participants at Pre-Test (*ns* = 22).

	Short Version						Long Version							
	Mozart		Albinoni		Control		Mozart		Albinoni		Control			
	M	*SD*	*M*	*SD*	*M*	*SD*	*M*	*SD*	*M*	*SD*	*M*	*SD*	*F*(5, 126)	*p*
**PRE-SCREENING**														
Age	69.77	3.25	69.68	3.01	69.32	2.98	68.59	3.10	69.46	3.31	69.50	2.92	< 1	0.842
Education	10.05	3.95	10.05	4.05	10.73	3.52	10.55	4.03	11.23	4.48	10.77	4.34	< 1	0.925
Vocabulary	42.86	11.95	40.46	12.27	45.32	10.75	39.50	10.43	41.05	9.60	38.64	12.31	< 1	0.438
PWB-PS	33.66	4.09	34.05	5.22	33.25	5.09	34.46	5.48	34.55	5.31	34.32	5.89	< 1	0.557
PANAS-P	32.02	4.94	32.14	6.57	31.09	5.22	33.89	6.42	32.5	6.97	32.93	6.55	1.27	0.282
PANAS-N	18.14	5.28	18.34	6.18	18.09	5.14	18.5	5.39	19.68	5.57	19.84	5.58	< 1	0.571
AFML	146.91	24.09	150.14	19.06	150.50	22.90	152.86	12.71	153.00	11.54	154.23	15.85	< 1	0.805
**COGNITION**														
Working Memory	2.73	1.24	2.59	1.10	2.50	0.96	2.32	0.57	2.82	1.01	2.64	1.00	< 1	0.640
Flexibility/Speed	118.50	20.11	117.96	30.74	119.82	23.61	122.05	31.60	123.73	32.03	124.09	35.98	< 1	.971
Verbal Fluency	11.91	4.02	13.05	3.48	12.46	3.85	12.55	3.84	12.68	4.01	12.68	3.84	< 1	0.878
Arithmetic	38.91	10.94	38.59	7.06	39.18	6.32	36.27	11.27	37.86	9.53	36.82	10.51	< 1	0.887
**EMOTION**														
Arousal	4.59	1.41	5.05	2.28	4.73	2.05	4.27	2.51	4.18	2.34	4.09	1.72	< 1	0.638
Mood	6.55	0.96	6.46	1.37	6.14	0.83	5.18	1.22	6.36	1.22	5.96	1.25	< 1	0.455
Dominance	6.64	1.40	7.18	1.33	6.91	2.09	7.05	1.68	6.59	1.62	6.91	1.27	< 1	0.807
